# Transition Toward Smart Hospitals: A Scoping Review of Features, Technologies, and Challenges

**DOI:** 10.1002/hsr2.71601

**Published:** 2025-11-30

**Authors:** Reza Rabiei, Wen‐Chin Hsu, Peivand Bastani, Sohrab Almasi, Saman Mortezaei, Shirin Dehghan

**Affiliations:** ^1^ Department of Health Information Technology and Management School of Allied Medical Sciences Shahid Beheshti University of Medical Sciences Tehran Iran; ^2^ Department of Information Management National Central University Taoyuan City Taiwan; ^3^ College of Business, Government and Law, Centre for Social Impact Flinders University Adelaide South Australia Australia; ^4^ Department of Health Information Technology School of Allied Medical Sciences Kermanshah University of Medical Sciences Kermanshah Iran; ^5^ Department of Health Information Management School of Allied Medical Sciences Tehran University of Medical Sciences Tehran Iran; ^6^ Vice Chancellor for Research Shiraz University of Medical Sciences Shiraz Iran

**Keywords:** digital hospital, digital transformation, intelligent hospital, smart hospital

## Abstract

**Background and Aims:**

The increasing elderly population, growing prevalence of chronic illnesses, and rising healthcare costs are driving healthcare providers to seek more affordable and cost‐effective care solutions. One promising approach is the integration of smart technologies within a hospital setting. This study aims to examine the transaction process toward smart hospitals and to explore the defining features, enabling technologies, and key challenges associated with digital and smart hospitals.

**Methods:**

This scoping review followed the Levac, Colquhoun, and O'Brien approach. Databases including IEEE, PubMed, Web of Science, ScienceDirect, Wiley, and Scopus were searched without any time restrictions to extract relevant articles, with the final search completed on September 11, 2025. Only articles published in English were included. A qualitative synthesis method and trend analysis were applied to investigate the emergence of digital and smart hospital concepts, along with the associated technologies, features, and challenges.

**Results:**

A total of 64 articles were included in this study. Assessing an organization's digital maturity is crucial for transitioning to smarter operations, encompassing structure, policies, governance, culture, and technology infrastructure. Key features of digital hospitals include the process of digitalization, system interoperability, and electronic medical records (EMRs). Transitioning to smart hospitals requires advanced technologies such as mobile networks, wireless systems, data analytics, location tracking, sensors, IoT, blockchain, and AI. The main benefits identified were improved technology adoption, stronger data management and security, enhanced organizational effectiveness, and greater digital health literacy. Additionally, six major challenges were highlighted, particularly around the adoption and integration of new technologies, data management and security, organizational barriers, and gaps in digital health literacy.

**Conclusion:**

Transitioning to digital processes and smart hospital operations can streamline workflows and improve clinical outcomes. However, due to the complexity and interdisciplinary nature of healthcare technology, setting digitalization strategies and making relevant policies are crucial. Successful adoption of a smart hospital system requires collaboration among healthcare providers, technology vendors, and other stakeholders.

**Abbreviations**
AI: artificial intelligence
ATC: Anatomical Therapeutic Chemical
CCMM: Continuity of Care Maturity Model
CRM: customer relationship management
EMR: electronic medical record
GPS: Global Positioning System
HIMSS: Healthcare Information and Management Systems Society
HIS: Hospital Information System
HISMM: Hospital Information System Maturity Model
ICD: International Classification of Disease
ICNP: International Classification for Nursing Practice
INFRAM: Infrastructure Adoption Model
IoT: Internet of Things
LIS: Laboratory Information Systems
LOINC: Logical Observation Identifiers Names and Codes
NLP: Natural Language Processing
PACS: Picture Archiving and Communication System
RFID: Radio Frequency IDentification
SCM: Supply Chain Management
SNOMED CT: Systematized Nomenclature of Medicine Clinical Terms
WBSN: Wireless Body Sensor Network

## 1 Introduction

Healthcare providers worldwide face numerous challenges, including an aging population, rising chronic disease prevalence, increasing costs, growing patient numbers, limited access to care, complex treatment options, and the need for greater patient engagement [1]. These pressures underscore the urgent need for innovation to enhance efficiency, quality, and sustainability in healthcare delivery [2]. Consequently, hospitals are moving from solely treating illnesses to managing overall patient health, increasingly adopting digital and smart hospital models to address these challenges [3]. Investments in information and communication technologies (ICTs) enhance management, clinical practice, and operations, while widespread technology adoption accelerates digital transformation [1, 3]. By converting analog data into digital formats, this transformation reduces costs and improves efficiency across healthcare, as well as in public and industrial sectors [4].

Digital transformation in healthcare aims to improve care quality, reduce costs, and leverage emerging technologies [5]. Technology enhances diagnosis, prevention, and treatment, while supporting evidence‐based clinical decisions [6].

Integrating technology into existing processes is essential for digital transformation [6, 7], which aims to enhance cross‐functional collaboration and coordination across an organization [8]. Digital transformation entails a fundamental shift, reshaping an organization's structure through information, communication, connectivity, and computing technologies [8]. In essence, it examines how the adoption of information technology can transform existing organizational frameworks [3].

### 1.1 Digitalization in Hospitals

There is no clear timeline for the digitization of healthcare. A study by Marques and Ferreira [9], reviewing 45 years of research, identified 7 main areas: integrated IT management, medical imaging, electronic medical records (EMRs), portable health devices, access to electronic health information, telemedicine, and data privacy. Digitization in hospitals began in 1973 with a patient management system covering identification, records, and statistics, followed by an integrated module for medical imaging [9]. Stephanie and Sharma's article notes that 1998 was a pivotal year for research, as it was when the term e‐health was first introduced. This review aims to explore digital health developments and understand the key issues related to healthcare delivery [10].

The recognized potential of information technology to improve healthcare has driven a global push for digitally advanced organizations, with hospitals implementing transformation programs [11, 12]. The concept of a digital hospital is multifaceted, influenced by factors such as the level of digitalization and quality of patient care. This concept refers to a healthcare facility where all processes—from patient records to diagnostics—are fully digital, often corresponding to Stage 7 of the HIMSS Electronic Medical Record Adoption Model (EMRAM), enabling patient‐centered care without time or location constraints [13, 14].

By digitalizing data and adopting systems such as order communication, image archiving, and EMRs, digital hospitals reduce costs, optimize resources, and accelerate treatment processes. Recent trends highlight a shift toward digital hospital management models [4]. Advances in ICT have also enabled the integration of wearable sensors with physical devices, supporting efficient data collection, processing, and large‐scale deployment [15]. The rapid growth of big data, network technologies, and AI has fueled a digital revolution in healthcare, giving rise to smart hospitals. Achieving a smart hospital is a central goal of healthcare digital transformation [16]. These hospitals integrate services such as diagnosis, treatment, management, and decision‐making, combining intelligent, informative, and digital healthcare principles [17, 18].

Establishing a smart hospital requires a comprehensive digital system that ensures fast access to information, supports evidence‐based decisions, and standardizes management [18]. A smart hospital integrates advanced technologies such as the Internet of Things (IoT), artificial intelligence (AI), and big data analytics to optimize processes, support real‐time decision‐making, and enhance patient safety through continuous monitoring and telemedicine [19, 20]. Its key features—continuous patient monitoring, advanced care, telemedicine integration, and improved diagnostics—further contribute to safer and more effective care [21]. Recent advances in wireless and mobile technologies have been crucial in realizing smart hospitals [22]. With advancements in care delivery, hospitals have shifted from treatment‐focused to patient‐centered care. Digital transformation is reshaping healthcare, giving rise to smart hospitals. Over the past decade, information technology has driven this ongoing transformation, continuously improving care quality. Digital and smart hospitals are the result of this evolution. Implementing a digital or smart hospital goes beyond just adopting emerging digital technologies; it requires a thorough understanding of all components and their interactions based on patient needs is essential; without it, progress is limited, and growing complexity can lead to inconsistency and dissatisfaction. Although existing studies have examined digital and smart hospitals—including their definitions, conditions, and technologies [23]—no comprehensive scoping review synthesizes the evolving concepts, processes, features, technologies, and challenges in this rapidly advancing field. Such a review is now critical, particularly given the accelerated adoption of digital solutions post‐COVID and the emergence of technologies like AI and IoT, which remain underexplored in prior reviews [24, 25]. This study aims to examine the transaction process toward smart hospitals and to explore the defining features, enabling technologies, and key challenges associated with digital and smart hospitals.

## 2 Methods

### 2.1 Design

The scoping review methodology aims to systematically identify and map all kinds of related and heterogeneous evidence, striving to determine the main aspects of a concept and provide a comprehensive overview of the existing literature [26, 27]. There are several frameworks for writing scoping review articles. According to the Joanna Briggs Institute methodology, these frameworks include Arksey and O'Malley's six‐step approach [26], Levac et al.'s method [28], and Peter et al.'s nine‐step approach [27]. This study followed the Levac, Colquhoun, and O'Brien approach [28] and was prepared in accordance with the PRISMA extension for Scoping Reviews (PRISMA‐ScR) (Supporting Information S1: File 1—PRISMA‐ScR Checklist) [29].

### 2.2 Research Goal and Question Clarification

In this study, researchers aimed to identify the steps needed to achieve a smart hospital, as well as the features, technologies, and challenges associated with digital and smart hospitals. The main questions guiding this study are:

1. What are the key steps and phases required to achieve a smart hospital?
2. What features and technologies are implemented in digital and smart hospitals?
3. What are the main challenges encountered during the digitalization and smart transformation of hospitals?

This formulation clarifies the study's objective and links the research questions directly to the overall aim of mapping the process and components of smart hospital implementation.

### 2.3 Breadth and Comprehensiveness of the Scoping Process

Articles were systematically retrieved from some of the world's largest academic databases, including IEEE, PubMed, Web of Science, ScienceDirect, Wiley, and Scopus. The search used a combination of MeSH terms and related keywords, such as (“digital hospital” OR “smart hospital” OR “intelligent hospital” OR “Digital transformation” OR “Digital innovation”) AND (characteristics OR requirement OR features OR challenges). The complete search strategy for each database is available in Supporting Information S2: File 2. These databases collectively host a vast array of literature, offering a broader range of journals compared to similar databases. The search covered an unrestricted timeframe and concluded on April 16, 2023, and the search was updated from May 1, 2023 to September 11, 2025. EndNote version 20.2.1 was used for the article selection and screening process.

### 2.4 Study Selection and Data Extraction

**Figure 1:**
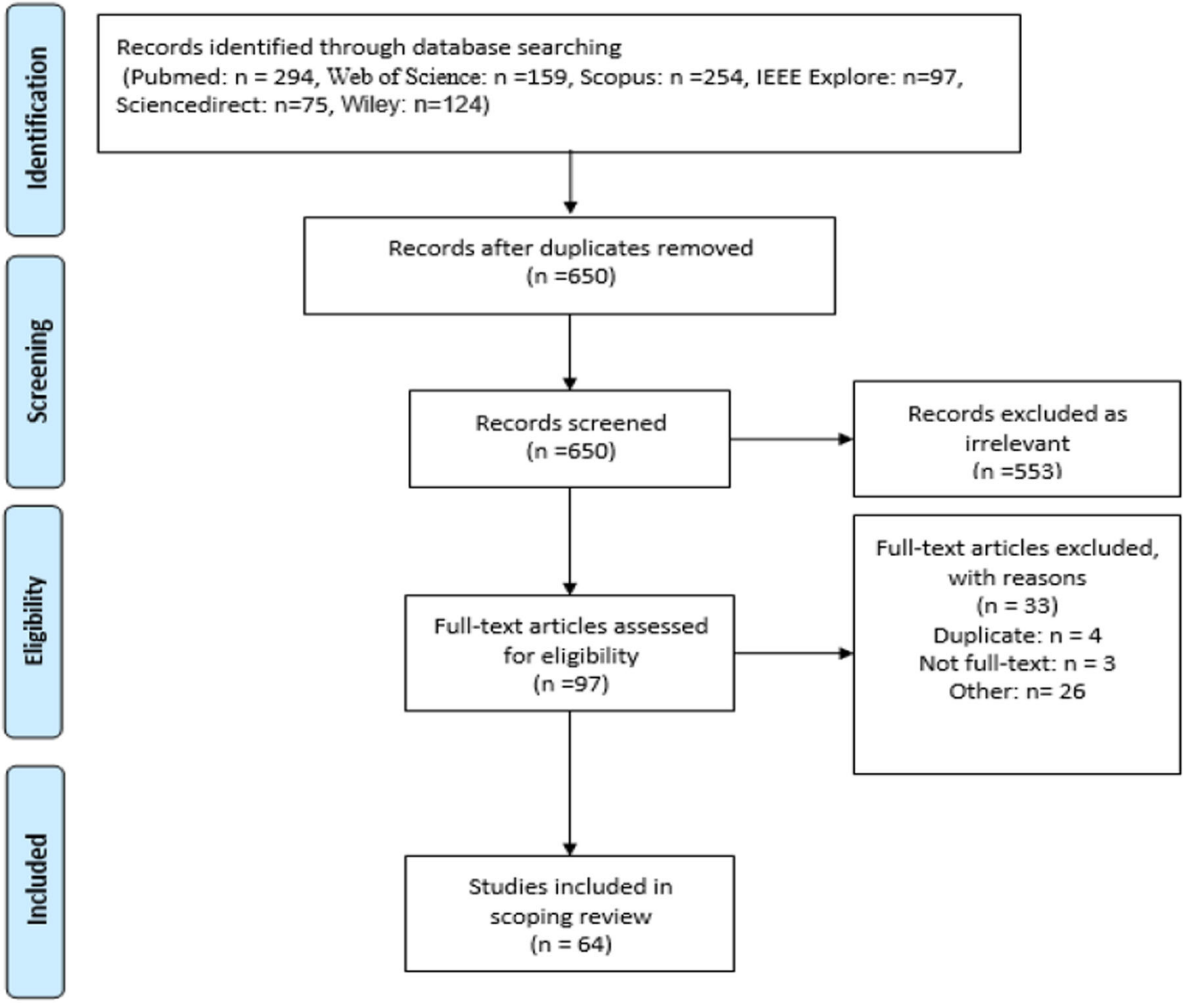
Visual representation of the paper selection process.

The retrieved articles were initially screened by title and abstract. Then, two authors (S.A. and S.D.) independently reviewed the articles, with any disagreements resolved by the senior author (R.R.). Once the senior author approved the screened articles, the full texts were reviewed by the two authors using the inclusion–exclusion criteria. Any remaining disagreements were resolved through discussion with the senior author. During the data extraction stage, the following information was extracted from each article: the first author's name, publication year, study location, objectives, main results, and challenges (Supporting Information S3: File 3—Characteristics of the included reviews). The article selection process is illustrated in Figure 1.

#### 2.4.1 Inclusion Criteria

1. Studies that described the features and technologies of digital/smart hospital.
2. Studies related to the implementation of digital and smart technologies in the hospital environment.
3. Studies that described the components and layers of the smart/digital hospital.
4. Studies related to the digital transformation process in the hospital.
5. Studies that were published in English.

#### 2.4.2 Exclusion Criteria

1. Studies related to the implication of information technology in hospital processes and user tasks.
2. Studies focused on the challenges of digitization and the use of smart technologies.
3. Studies published in languages other than English.
4. Studies that were review articles, letters to the editor, note, editorials, or book chapters.
5. Studies where the full text was not available.

### 2.5 Quantitative and Qualitative Data Analysis

**Figure 2:**
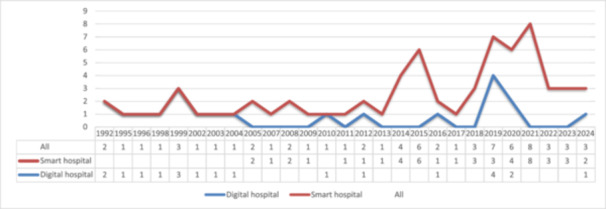
Chronological distribution of the included articles.

**Figure 3:**
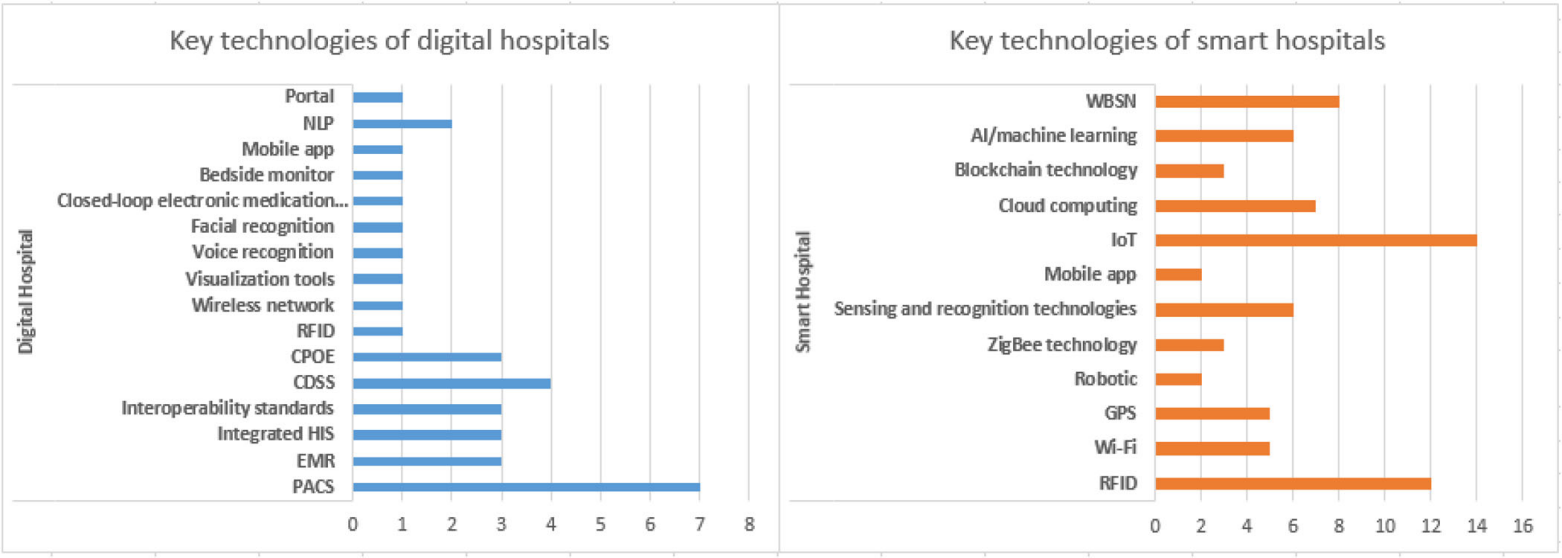
Key technology in digital/smart hospital.

At this stage, the bibliographic information of the reviewed articles, such as the publication year, country, and publication type (conference or journal), was extracted and entered Excel (version 16) for analysis. This task was performed by one of the authors (S. A.). Trend analysis was used to investigate the emergence of digital and smart hospital concepts and the technologies associated with these concepts (Figure 2). Content analysis was employed to identify the features (Tables 1 and 2), technologies (Tables 3 and 4, Figure 3), and challenges (Table 5) of each digital and smart hospital.

**Table 1:** Key features of digital hospitals.

Main themes		Subthemes	Quotes	References
Interoperability of systems	Inside hospital	Sharing data Data integration	“…technical functionalities to wider digital transformation capabilities” “…Management intelligence through digital health data…”	Krasuska et al. [12]; Hu et al. [30]; Yoo et al. [31]; Chang et al. [32]
	Outside hospital	Sharing data Integration across ecosystems Effective interaction among different components of the healthcare system	“…exchange of information and communication beyond an individual hospital…” “…Exchange of prescription information in a structured way…” “…data exchange and sharing platforms…”	Krasuska et al. [12]
Digitalization of all information		Digital format conversion (paperless) Analog to digital process	“…Digital hospital is moving towards paperless age gradually…” “… Establishing PACS, radiology digital reading center…”	Hu et al.[30]; Yoo et al. [31]; Ranschaert [33]; Liu et al. [34]
Provision of stable core digital infrastructure		Direct care support Administrative and financial applications Management information systems Using data retrieval and analysis, backup, clinical data warehouse, disaster recovery Information system infrastructure Communication with patients Integrating with systems outside hospital Customer relationship management Health information exchange	“…Digital hospital is moving towards paperless age gradually by electronic prescriptions, EMR, electronic requisitions, electronic reporting, electronic office…” “… composed of three application domains, i.e., the core application, information infrastructure, and channel domain,…” “… direct care, support, administrative and financial, and management information system,…”	Kim et al. [4]; Hu et al. [30]; Yoo et al. [31]; Chang et al. [32]; Yoo et al. [35]
Interconnected clinical information systems		Hospital Resources Plan PACS LIS EMR SCM CRM Office Automation Decision Support System Tele‐medicine system Wireless Application Systems Closed‐loop medication administration	“ … Set up a data center to store and exchange data in multiple application systems…” “… close‐loop medication administration (CLMA), clinical data warehouse (CDW), health information exchange (HIE), and disaster recovery (DR)…” “…information infrastructure domain of the system includes applications,…” “…communication interface standardization, function modularisation, common sharing of medical information…” “…HIS, RIS and PACS in consistence with DICOM, HL7 and TC251 standards…” “…Applications for user‐centered EMR and CDS, CLMA, mobile EMR, and dashboard system for care coordination…”	Kim et al. [4]; Hu et al. [30]; Yoo et al. [31]; Chang et al. [32]; Yoo et al. [35]; Takeda et al. [36]; Soman et al. [37]

**Table 2:** Key features of smart hospitals.

Main themes	Subthemes	Quotes	References
Mobile integrated solutions	Communication and collaboration among healthcare professionals Postdischarge acute care visits Virtual visit Communication with a healthcare provider	“…Using mobile technologies to support the delivery of care outside traditional settings and closer to home…” “…measurement, diagnosis, prevention, monitoring, and treatment at the personal and community level…” “…smart health applications (apps) included; Smart Clinic, Smart Patients, and Smart Hospital…”	Coronato and Esposito [21]; Thakare and Khire [38]; Park et al. [39]; Lee and Kim [40]; Coronato et al. [41]; Vecchia et al. [42]
Wireless network	Wireless connections Remote patient monitoring Linking healthcare providers, managerial and operational staff, patients, visitors, and hospital equipment	“…RFID based conceptual frameworks or smart hospital management system…” “…The RFID systems can be used to manage medicine and hospital equipment, track patients care, and help doctors…” “…These sensor (WBANs) nodes can be worn externally or implanted internally in the human body to monitor multiple health parameters of patients…”	Uslu et al. [11]; Lee and Kim [40]; Coronato et al. [41]; Vecchia et al. [42]; Sutherland et al. [43]; Wu et al. [44]; Gomez‐Sacristan et al. [45]; Mahmood et al. [46]; Huang et al. [47]; Thangaraj et al. [48]; Venkateswari and Rani [49]; Alharbe and Atkins [50]; Jang et al. [51]; Pan et al. [52]
Data analytics	Using data for effective decision‐making Automatic information summarization Expanding public health surveillance through detecting identification of disease patterns Detecting anomalies and providing treatment to patients Early disease detection	“…Passive RFID and/or combined with ZigBee technology could be used to improve healthcare organisations through continuous data collection, supporting real‐time decision‐making…” “…A Patient remote monitoring system includes wearable devices which are developed by using Internet of Things…”	Alharbe and Atkins [50]; Alharbe et al. [53]; Rizwan et al. [54]
Remote care system	Remote patient monitoring Telemedicine service. Constant monitoring of the patient′s condition Control check‐ups outside medical facilities	“…future in which providers and hospitals transition medical care delivery to the home…” “…Centralized services housed in Cloud architectures or telemedicine/tele‐assistance services…”	Gomez‐Sacristan et al. [45]; Pham et al. [55]; Rosen et al. [56]
Smart technical infrastructure	Interconnected IoT and digital features Using cloud‐based servers, big data and cloud computing for all data processing Various applications used for all the various systems and operations in the hospital. Utilizing Sensors, connection methods, internet protocols, databases, cloud computing, and analytic as infrastructure	“…infrastructure relies on the integration of Radio Frequency IDentification (RFID) and photosensor technologies to identify, locate and track clinicians and patients equipped…” “…Smart Hospital Management Information System (SHMIS) that allows the dynamic control of objects and transforms operational processes…” “…infrastructure consists in a Service Oriented Architecture and exhibits a set of services and components…” “…Internet of Things (IOT) Enabled Smart Autonomous Hospital Management System…”	Alharbe and Atkins [50]; Alharbe et al. [53]; Kumar and Suresh [57]
Location, recognition, tracking, and identification systems	Detecting, locating, and monitoring patients Tracking assets and equipment with modern sensor‐based technologies in a real‐time environment	“…semantic integration of different positioning systems…” “…Radio Frequency IDentification (RFID) and ZigBee technologies has made it possible to identify, locate, and track objects in various environments in real‐time” “…Object (patients, staff and assets) movement monitoring is required for recording purposes and for security measures…”	Yao et al. [3]; Uslu et al. [11]; Coronato and Esposito [21]; Lee and Kim [40]; Coronato et al. [41]; Vecchia et al. [42]; Sutherland et al. [43]; Wu et al. [44]; Mahmood et al. [46]; Huang et al. [47]; Alharbe and Atkins [50]; Alharbe et al. [53]; Rizwan et al. [54]; Alharbe and Atkins [58]; Yamashita et al. [59]
Near‐real‐time access to data	Informing and accelerating decisions, deliver more‐personalized care, and lowering costs Enhancing the transparency of resource utilization and better management of fluctuating caseloads	“…Radio Frequency IDentification (RFID) and ZigBee technologies can be used together, to provide real‐time information for decision support and to create a secure…” “…Real‐time access to data, by providing an up‐to‐date database…”	Wu et al. [44]; Gomez‐Sacristan et al. [45]; Alharbe and Atkins [50]; Alharbe et al. [53]; Alharbe and Atkins [58]
Workflow automation	– Making more free time for staff to devote more time to direct patient care – Improving efficiency of many back‐office and front‐office processes	“…smart hospital management system that can be used to detect, locate, and monitor patients and track assets and equipment using modern sensor technologies…” “…wireless communication technology that can be used as an automatic identification technology…”	Mahmood et al. [46]; Alharbe and Atkins [58]
Learning health systems	– Virtual reality training for clinicians	“…use of simulation tools allows this task to be undertaken and using different hypotheses…”	Gomez‐Sacristan et al. [45]

**Table 3:** Key technologies of digital hospitals.

Technologies	Applications		References
PACS	Securely storing and digitally transmitting digital images and clinically relevant reports Transmission of medical images between healthcare providers and remote specialist		Chang et al. [32]; Ranschaert [33]; Yoo et al. [35]; Hruby et al. [60]; Kuzmak and Dayhoff [61]; Siegel et al. [62]; Pavlopoulos and Delopoulos [63]
EMR	Central storage Processing exchanging and updating medical information through internet and wireless network		Krasuska et al. [12]; Yoo et al. [31]; Takeda et al. [36]
Integrated HIS	Real‐time documentation and cross‐checking of care practices Providing virtual desktop infrastructure, mobile EMR system, and large touchscreen‐based dashboard system to healthcare providers Patient participation and engagement using mobile technologies		Hu et al. [30]; Yoo et al. [31, 35]
Interoperability standards	Information Exchange standards (HL7) Content standards (SNOMED CT, ICD‐10, ICNP, ATC, LOINC) Privacy and security standards Functional standards (work processes, workflow, and dataflow models)		Hu et al. [30]; Yoo et al. [31, 35]
CDSS	Early and accurate diagnosis Identifying high‐risk patients Preventing medical errors, Providing up‐to‐date information to healthcare providers Providing patient care remotely Providing standardizing care Generating patient‐specific recommendations Integration into electronic medical records		Krasuska et al. [12]; Hu et al. [30]; Yoo et al. [31, 35]
CPOE	Fewer medications errors and adverse drug events Greater guideline adherence Improved disease control Decreased dispensing turnaround times		Yoo et al. [31, 35]; Rizwan et al. [54]
RFID	Matching the medication and patient with the physicians' order information		Krasuska et al. [12]
Wireless network	Tracking the patient conditions by laptops, table PC, PDA No time and place constraints Improving diagnosis and treatment efficiency		Hu et al. [30]
Visualization tools	Advanced analytics capability to support the move from reactive to proactive/predictive models of care		Kim et al. [4]
Voice recognition	Automatic EMRs entry Management and documentation of medical activities through data linking between medical devices		Kim et al. [4]
Facial recognition	Contactless facial recognition system for infection prevention among medical personnel instead of physical contact Supporting patient identification		Kim et al. [4]
Closed‐loop electronic medication management	Electronic prescription with technology‐assisted identification		Krasuska et al. [12]
Bedside monitor	Providing Hi‐Chart (illustration of tests and procedures using animated videos) Providing customized services to patients (hospital stay guidance, disease education, medical record requests, convenience services)		Kim et al. [4]
Mobile app	Patients	Making an appointment on mobile app Communications among patients and healthcare providers via diverse of media channels Using mobile devices to enable care provision in nontraditional and home‐based settings	Kim et al. [4]
	Staff	– Communication‐enabled multidisciplinary interventions and assessments	Kim et al. [4]
NLP	Automated detection of a specified clinical event through free‐text input Clinical decision support based on question answering Offering effective care to individuals with different speech impairments		König et al. [1]; Krasuska et al. [12]
Portal	Faster access to health data for patients and families Consistent and continuous communication between patients and the care team Improved efficiency of nursing services and treatment follow‐up, especially in nurse‐led models		Hughes et al. [64]

**Table 4:** Key technologies of smart hospitals.

Technologies	Applications		References
RFID	Managing hospital equipment Tracking patients care Checking patient–drug compliance Monitoring patients regularly and remotely by healthcare providers		Yao et al. [3]; Coronato and Esposito [21]; Lee and Kim [40]; Coronato et al. [41]; Vecchia et al. [42]; Sutherland et al. [43]; Mahmood et al. [46]; Huang et al. [47]; Alharbe and Atkins [50]; Alharbe et al. [53]; Rizwan et al. [54]; Alharbe and Atkins [58]
Wi‐Fi	For a variety of sensing applications, such as gesture recognition and fall detection A driving factor in equipping hospital to technologies like blockchain, biotelemetry, drug development		Coronato and Esposito [21, 65]; Coronato et al. [41]; Gomez‐Sacristan et al. [45]; Alharbe et al. [53]; Chen et al. [65]
GPS	Tracking patients in emergency conditions		Coronato et al. [41]; Huang et al. [47]; Yamashita et al. [59]; Hughes et al. [64]; Peleato et al. [66]
Robotic	Transporting medical equipment, medicine, food Performing medical actions on behalf of humans (surgery, rehabilitation, nursing care, logistics)		Peleato et al. [66]; Vazquez‐Santacruz et al. [67]
ZigBee technology	Monitoring patients' movements Monitoring patients and room temperature Accessing real‐time medical data Continuous patient statistics monitoring (such as body temperature and blood pressure to send an emergency alarm message or SMS to staff in charge)		Alharbe and Atkins [50]; Alharbe et al. [53]; Alharbe and Atkins [58]
Sensing and recognition technologies	Wearable sensors	– Electrocardiogram sensor (ECG)	Uslu et al. [11]; Atta [68]
		LDR	
		GPS	
		Blood pressure cuff	
		Heartbeat Sensor	
		Physiological sensors	
		– Spirometer	
	Ambient sensors	– Temperature sensor (LM35)	Uslu et al. [11]; Zhang et al. [69]
		Light dependent	
		Thermometer	
		Hygrometer	
		Noise detector	
		Humidity sensor	
		– Motion detector	
	Location sensors	– Infrared	Uslu et al. [68]; Atta [68]
		Zigbee	
		Active RFID	
		– Binary sensors (Window contact, Door contact, Light switch, Remote control switch)	
Mobile app	Alarms and reminders on taking medications on time, latest trends on recovery, payment information		Park et al. [39]; Jebamani et al. [70]
IoT	Tracking real‐time location of medical equipment Monitoring patients' health Remote patient monitoring		Uslu et al. [11]; Canfell et al. [14]; Dhariwal and Mehta [17]; Thangaraj et al. [48]; Alharbe et al. [53]; Rizwan et al. [54]; Kumar and Suresh [57]; Zhang et al. [69]; Fischer et al. [71]; Mezenner et al. [72]; Akbarzadeh et al. [73]; Ravali and Priya [74]; Rodrigues et al. [75]; Sakthikumar et al. [76]
Cloud computing	Providing safe, secure, and real‐time data sharing with relevant doctors and care providers		Gomez‐Sacristan et al. [45]; Alharbe et al. [53]; Kumar and Suresh [57]; Zhang et al. [69]; Rodrigues et al. [75]; Sakthikumar et al. [76]; Talib et al. [77]
Blockchain technology	Authorization among system users Remote monitoring of patient vital signs Decentralized record management Data distribution		Canfell et al. [14]; Pham et al. [55]; Kiania et al. [78]
AI/machine learning	Facilitating fast, frictionless decision‐making Focusing on prevention, monitoring, and care delivery Autonomous monitoring aids in the detection of anomalies and trends in real‐time		Dhariwal and Mehta [17]; Kumar and Suresh [57]; Peleato et al. [66]; Talib et al. [77]; Rajakumari and Madhunisha [79]; Mi et al. [80]
WBSN	Capturing patient data and route them for processing Monitoring the vital signs		Lee and Kim [40]; Sutherland et al. [43]; Huang et al. [47]; Alharbe and Atkins [50]; Jang et al. [51]; Alharbe et al. [53]; Rizwan et al. [54]; Rodrigues et al. [75]

**Table 5:** Challenges of the digital/smart hospital.

Main themes	Subthemes	Quotes	Frequency (percentage)	References
Technology adoption and integration	Functional and nonfunctional system issues	“…need to develop PHR functionalities that are meaningful to both patients and doctors…” “The protocol parameters could be further optimized to provide lower delay…”	22 (38%)	Yoo et al. [31]; Thakare and Khire [38]; Vecchia et al. [42]; Alharbe and Atkins [50]; Jang et al. [51]; Rizwan et al. [54]; Pham et al. [55]; Alharbe and Atkins [58]; Vazquez‐Santacruz et al. [67]; Rodrigues et al. [75]; Aktepe et al. [81]; Scott et al. [82]
	Interoperability issues	**“…**The full integration of RIS and PACS systems has not been achieved” “…Lack of semantic integration of different types of positioning technologies…” “…system needs the seamless connection among HIS, RIS and PACS through modularized interfaces, achieving system function…”		Kim et al. [4]; Krasuska et al. [12]; Coronato and Esposito [21]; Hu et al. [30]; Chang et al. [32]; Soman et al. [37]; Wu et al. [44]; Mahmood et al. [46]; Jang et al. [51]; Kumar and Suresh [57]; Sakthikumar et al. [76]
	Lack of data terminology and standards	**“…**the importance of development of standards for integrating clinical information systems” “Standardization of communication between IoT devices in smart hospitals”		Uslu et al. [11]; Davidson and Chismar [83]; Williams et al. [84]; Barnett et al. [85]
	Poor data quality	**“…**Modeling medical knowledge with RFID and CEP is complicated, as medical knowledge is often fuzzy and hard to verify for accuracy…” “…need to acquire Accurate and reliable data collection from sensors to meet medical standards…”		Yao et al. [3]; Atta [68]; Rajakumari and Madhunisha [79]
Data management and security	Privacy and security issues Data governance and ethical concerns	**“…**healthcare system handles a significant amount of confidential personal information. Inappropriate gathering, deliberate misuse, and unauthorized entry…” “…The legal aspects of data protection for integrated digital systems are still unclear” “…Security and privacy challenges in connecting numerous IoT devices in smart hospitals…” “… improving data security and user access control for PACS systems” “The problems of privacy and security issues for the electronic patient record system have not been solved yet”	12 (20%)	Kim et al. [4]; Canfell et al. [14]; Hu et al. [30]; Yoo et al. [31]; Liu et al. [34]; Takeda et al. [36]; Vecchia et al. [42]; Huang et al. [47]; Alharbe et al. [53]; Siegel et al. [62]; Pavlopoulos and Delopoulos [63]; Zhang et al. [69]; Sakthikumar et al. [76]; Mi et al. [80]; Wischnevsky and Damanpour [86]; Cresswell et al. [87]
	legal and regulatory framework			
Organizational challenges	Organizational structure and reediness Lack of management support and poor leadership Rigid policies and procedures	**“…**Availability of necessary resources is crucial for effectively implementing smart healthcare strategies” “…Lack of support for location reasoning, and lack of semantic integration of different positioning technologies” “…hospitals are more or less competitive with each other, and due to the lack of effective leadership, precious experiences…”	9 (15%)	Kim et al. [4]; Tricco et al. [29]; Park et al. [39]; Coronato et al. [41]; Gomez‐Sacristan et al. [45]; Huang et al. [47]; Peleato et al. [66]; Zhang et al. [69]; Fischer et al. [71]
Cost	High costs	**“…**adoption of smart health strategies involves expenses related to acquiring infrastructure, hardware…” “…expensive processes for searching and detecting landmarks in current methods…”	7 (12%)	Liu et al. [34]; Soman et al. [37]; Alharbe and Atkins [58]; Siegel et al. [62]; Peleato et al. [66]; Jebamani et al. [70]; Ravali and Priya [74]
Digital health literacy	Resistance to change End‐user behavior	**“**The changing nature of the IS profession in the healthcare industry**”** **“…**User acceptance and understanding of the centralized PACS system by medical staff is needed”	5 (1%)	Yoo et al. [35]; Pavlopoulos and Delopoulos [63]; Davidson and Chismar [83]
	Poor computer skills Lack of training and support Lack of knowledge			Liu et al. [34]; Rodrigues et al. [75]
Changing user work practice and workflows	Disrupted workflows and reduced productivity	**“…** implementation of new smart healthcare strategies can cause interruptions in the organization's usual workflows and processes” “Develop new access rules and policies for the EPR system and telemedicine environment, building on the existing rules for the hospital information system”	3 (0.05%)	Levac et al. [28]; Takeda et al. [36]; Yamashita et al. [59]

**Figure 4:**
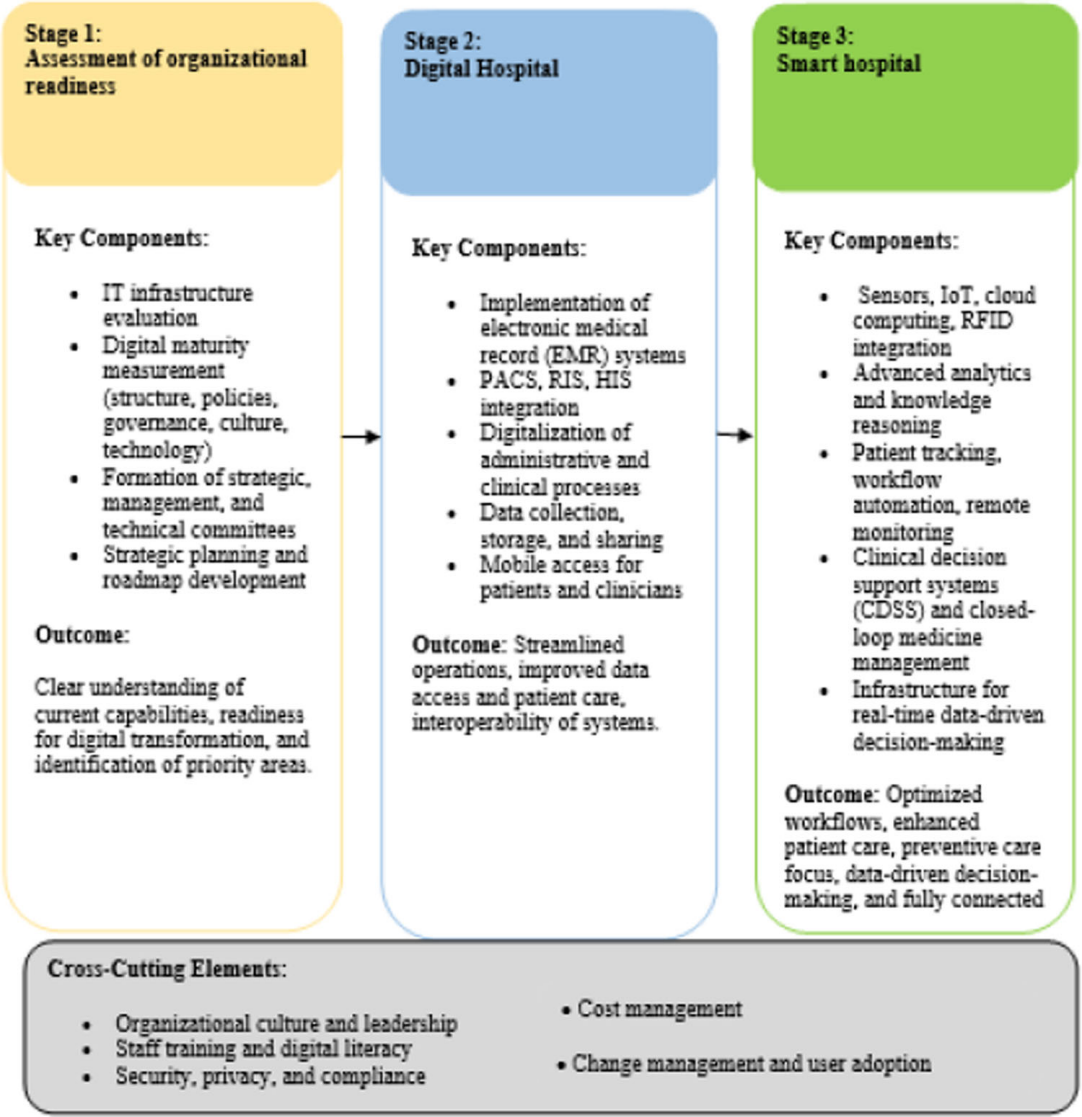
Evolutionary framework for hospital digital transformation: from readiness to smart hospital.

To conduct this analysis, a thematic approach was used. The text of the articles was examined to extract codes related to features and technologies. These codes were then reviewed and categorizing to create new concepts. For the qualitative thematic analysis [88], the findings of the studies were examined line‐by‐line to extract primary codes based on the research purpose and question. After extracting and re‐examining the primary codes, final codes were identified. These final codes were then categorized and classified to create subthemes and main themes, involving more complex combinations. The subthemes and themes were finalized after a thorough review. Descriptive statistics, including frequencies and percentages, were used to summarize the characteristics of the included studies. Specifically, the geographical distribution of studies and the type of publication (journal vs. conference) were calculated. All quantitative data were managed and analyzed using Microsoft Excel (version 16). Finally, we propose an evolutionary framework for hospital digital transformation, encompassing all stages from organizational readiness to the development of a fully smart hospital (Figure 4).

### 2.6 Implications

This study highlights the differences between digital and smart hospitals, outlining the phases involved in transitioning to a smart hospital and detailing the specific technologies and attributes of each. Policymakers, hospital administrators, and researchers in this field can obtain valuable insights from these findings.

## 3 Results

A total of 1003 articles were retrieved in this study, 353 of which were removed due to duplication. Out of the remaining 650 articles, 553 were excluded after reviewing their titles and abstracts, leaving 97 articles. After the title and abstract screening, the full texts of relevant articles were assessed against the inclusion and exclusion criteria, resulting in 64 articles for the final review.

### 3.1 Key Features of the Included Studies

The geographical distribution of the studies revealed that most came from India (*N* = 11,17%), the United States (*N* = 10, 15%), South Korea (*N* = 7, 11%), and China (*N* = 7, 9%). Most articles were published in Journals (*N* = 43, 67%), while the rest were presented at conferences (*N* = 21, 33%).

The number of published articles has steadily increased since 2014. Trend analysis revealed that discussion about the digitalization of hospitals in radiology began with the implementation of Picture Archiving and Communication Systems (PACS) [33, 60, 61, 62]. Later, the electronic patient record system was introduced [36]. The concept of smart hospital started in 2005 with the adoption of mobile/wireless technologies, Radio Frequency IDentification (RFID), and service‐oriented architectures [43, 44].

### 3.2 Steps to Create a Smart Hospital

The literature review highlights the key steps to establishing a smart hospital. First, assessing readiness is crucial for laying the groundwork and setting up the right infrastructure. Next, digitalizing process is vital to streamline operations. Overall, creating a smart hospital typically involves three pivotal stages as follows.

### 3.3 First Stage: Assessment of Organizational Readiness

To move toward digitalizing its process, an organization needs a clear understanding of its IT infrastructure [84]. Measuring the level of digital maturity is essential for assessing the organization's state of digital transformation, including its structure, policies, governance, culture, and technology infrastructure [82]. This measurement is the first step in the digital transformation journey, helping to determine the scope and extent of digitalization.

Once the digital maturity is assessed, strategic, management, and technical committees are established, and roles and responsibilities are assigned. The next step is to develop a strategic plan and roadmap that outlines the governance structure and top‐priority projects for implementing digital transformation [82, 84, 85].

Several frameworks have been suggested to evaluate an organization's readiness and measure its level of digital maturity. These include the HIMSS EMRAM, the HIMSS Analytics Infrastructure Adoption Model (INFRAM), and the Continuity of Care Maturity Model (CCMM) [4, 84].

Scott and colleagues proposed a checklist to evaluate hospitals' readiness for digital transformation, which includes 19 questions: 13 related to EMR implementation and 6 to digital transformation more broadly. The checklist covers several areas in the first set of questions.

#### 3.3.1 Organizational

Leadership, governance, change leaders, and implementation plan.

#### 3.3.2 Technical

Vendor selection, information technology, project management teams, system and hardware alignment with clinician workflows, and interoperability with legacy systems.

#### 3.3.3 Training

User training, post‐go‐live contingency plans, rollout sequence, and staff support at the point of care.

The second set of questions addresses as follows.

#### 3.3.4 Cultural

Clinically focused vision statement and communication strategy, readiness for change surveys.

#### 3.3.5 Digital Disruption Syndrome Management

Managing the effects of digital disruption.

#### 3.3.6 Patient Care Improvement

Post‐go‐live system optimization, quality and benefit evaluation, and ongoing digital innovation [82].

A study by Krasuska and colleagues examined how organizational culture, workforce, and digital strategy and infrastructure contribute to digital transformation [12]. Carvalho et al. [89] highlighted the crucial role of technological infrastructure in hospitals, including the hospital information system, as a foundation for digital transformation. Their study identified key components of the Hospital Information System Maturity Model (HISMM), such as data analysis, strategy, personnel, EMR, information security, and IT infrastructure [89].

### 3.4 Second Stage: Digital Hospital; Features and Technology

Digitalization is the second step in the journey toward digital transformation. Establishing a digital hospital is a dynamic, long‐term process that involves integrating various systems. The first requirement for creating a digital hospital is a setting up a robust digital infrastructure. This core infrastructure includes applications for clinical and administrative tasks, a solid information infrastructure, and systems that can work together seamlessly [30].

The literature review revealed that hospitals started their digital transformation by adopting PACS for storing and sharing radiology images with the Radiology Information System (RIS) and the Hospital Information System (HIS) [31, 33, 60, 61, 62]. As hospitals move toward digitalization, they replace traditional paper‐based systems to enhance efficiency. The initial steps typically involve digitalizing administrative systems or implementing a comprehensive EMR system [30, 36].

Digitalization helps healthcare providers improve patient care through electronic data collection, storage, and sharing, which streamlines interactions between healthcare providers [30]. In a digital hospital, the healthcare team can conveniently access a patient's medical history, imaging and test results using computers, tablets, or smartphones, rather than paper files. Transferring radiology images through PACS using the Digital Imaging and Communications (DICOM) standard is a key example of information flow in digital hospitals [32].

Patients can also use mobile technology to communicate with providers, make appointments, and view their medical information [4]. Health data collection and exchange must be comprehensive and accurate. Standards of exchange, including those for structural, content, and security, are necessary to ensure data compatibility, analysis, extraction, and utilization [30].

Additionally, bedside monitoring technologies automatically record data including, but not limited to, vital signs such as heart rate, blood pressure, and body temperature directly into the patient's EMRs. In a digital hospital, RFID tags are used for patient identification, allowing quick access to their vital signs, medications, and radiology images. By scanning the barcode, nurses can access all medical information online and verify medications against the physicians' order, preventing drug prescriptions errors [31].

Closed‐loop electronic medicine management, combined with Computerized Physician Order Entry (CPOE) and RFID, collaborates with various Clinical Decision Support Systems (CDSS) to mitigate drug interactions and errors. This integration prevents redundant drug orders and effectively manages prescriptions [31]. Digital hospitals focus on patient care [4], with core features including interoperability, data digitalization, and integrated EMR systems [30]. The goal of digitalizing data at this stage is to create a digital infrastructure and integrated clinical information systems that can interact seamlessly [4, 30, 31, 32, 35].

### 3.5 Third Stage: Smart Hospital; Features and Technology

A smart hospital represents the next evolution beyond a digital hospital. It goes beyond just having connected devices on a high‐speed infrastructure network [45]. Smart hospitals use advanced technologies to improve department management, streamline clinicians' workflows, and improve patient care. Transforming a hospital into a smart facility offers significant advantages, including cost reduction, better treatment outcomes, and improved planning. This transformation involves careful needs assessment, strategic equipment acquisition, and seamless device integration and installation. Simply moving from paper‐based to digital workflows is only a part of the entire process; the goal is to improve data collection and analysis to enhance decision‐making.

In smart hospitals, the focus extends beyond treatment, prevention, and health, with a collaborative approach to continuity of patient care instead of episodic treatments [38, 45], prevention, and patient self‐care [39, 70]. Alharbe et al. [53] presented a six‐layer architecture for implementing a smart hospital, consisting of:

*A data processing layer*: A network of sensors to collect real‐time data;

*Data integration layer*: Organizes and stores the data collected from the first layer;

*Cloud computing layer*: Increases resource sharing capacity through the internet;

*Network structure layer*: Transfers and shares data using technologies such as ZigBee, Wireless Local Area Networks (WLAN), Cellular Mobile Networks, and Personal Area Networks (PANs);

*Knowledge reasoning layer:* Processes and extracts knowledge from the data set; and

*Visualization layer*: Improves information presentation using visual tools [53].

Uslu et al. [11] suggested a five‐layer architecture for developing a smart hospital using IoT technology. These five layers include:

*Sensing layer*: Involves data collection technologies that gather or update structural and nonstructural information created or made available by the system;

*Networking layer*: Facilitates data transfer to remote servers and connects systems and platforms;

*Remote servers layer*: Encompasses the remote computing infrastructure of the IoT system;

*Knowledge layer*: Includes IoT's intelligent analysis and decision‐making module, where knowledge processing takes place; and

*Applications layer*: Consists of service platforms used by the system's users [11].

Both architectures aim to structure smart hospital systems to enhance patient care, data management, and operational efficiency. Alharbe and Atkins's six‐layer model emphasizes a hierarchical separation of data processing, integration, cloud computing, networking, knowledge reasoning, and visualization, providing detailed clarity and strong support for data‐driven decision‐making; however, its complexity may increase implementation challenges and resource requirements [50]. Uslu et al.'s five‐layer IoT‐based model focuses on sensing, networking, remote servers, knowledge processing, and applications, offering a more streamlined structure that facilitates IoT integration and real‐time responsiveness, though it gives less explicit emphasis to visualization and knowledge reasoning compared to Alharbe's model [11]. Overall, both models share the goal of improving hospital intelligence and connectivity, but the choice between them may depend on the institution's priorities: detailed data processing and visualization versus simplified IoT integration and faster deployment. In smart hospitals, a wireless network continuously collects essential information from various users in different locations. An intelligent cloud computing system, including various sensors, such as wireless physical sensors and a data collection layer, enables autonomous data reception and transmission upon user demand [53].

Integrating IoT, sensor technology, and cloud technologies into smart hospitals address recourse limitations by extending coverage to large geographical areas and improving accessibility for multiple users. Additionally, the development of smart hospitals relies on infrastructure‐architecture approaches, service‐oriented architecture, and software development and delivery models [53].

With the rise of data digitalization and improved system interactions, data security has become a crucial concern. Blockchain technology, as suggested by various studies, offers a promising solution to this issue [14, 55, 78]. Previous studies have shown that RFID technology excels in providing safer and more efficient services. As a wireless sensor‐based technology, RFID significantly enhances interaction with hospital information systems [46].

RFID technology extends its application in smart hospitals beyond medication administration. It interacts with other technologies, such as sensors and wireless networks to aid in patient and personnel tracking, equipment and device management, patient identification, drug inventory, hospital equipment inventory, patient care tracking, and remote patient monitoring [46, 47].

Creating a smart hospital, rather than merely a digital hospital, requires more than just data digitalization. It involves a transformation in the organization's mindset, values, and strategy. This shift necessitates restructuring current processes and developing a smart infrastructure within the hospital. A smart hospital aims to revolutionize care delivery processes, management systems, and even physical structures to introduce an innovative approach to patient care.

### 3.6 Challenges of the Digital/Smart Hospital

A total of 17 challenges for digitization and adopting smart technologies in hospitals were initially identified. After merging similar and repeated issues, six main challenges remained. The most important challenge was technology adoption and integration, which included concerns about data quality, lack of standards and terminology, interoperability, and the capabilities and features of the technology (*n* = 22, 38%). Besides technology‐related challenges, the other major issues were data management and security (*n* = 12, 20%), organizational challenges (*n* = 9, 15%), and cost (*n* = 7, 12%).

## 4 Discussion

This study outlined the process of achieving a smart hospital and detailed the features and technologies available in both digital and smart hospitals. However, the first step in implementing these technologies is measuring the digital maturity of the organization to determine its readiness for digital transformation. Digital transformation entails a radical shift in strategy, organizational structure, and power distribution within healthcare organizations [90]. Therefore, organizations must restructure their strategy and organizational structure, which can be a complex learning process for leaders and the organization as a whole [6].

### 4.1 Assessment of Organizational Readiness

Various models such as HIMSS EMRAM, INFRAM, and CCMM have been widely used to assess digital maturity in healthcare organizations. While these models provide structured frameworks, they predominantly emphasize technological aspects, often overlooking organizational and human capabilities. For example, the HIMSS EMRAM model focuses on data exchange within the organization but has limitations regarding interoperability with external health‐related organizations, which is crucial for effective public health management [91]. This technology‐centric focus may lead to incomplete assessments of a hospital's readiness to adopt advanced digital solutions, as successful implementation also relies heavily on management, workforce skills, and organizational alignment. Several studies highlight the importance of these nontechnological dimensions. Yilma et al. [92] emphasized that management, financial, operational, and technical capabilities, as well as budget allocation and organizational alignment, are critical for improving readiness for EMR implementation. They also stressed the need for targeted training programs and initiatives to enhance the knowledge and attitudes of healthcare providers toward EMRs [92]. Similarly, Tun and Madanian identified six key categories necessary for clinical information system implementation in developing countries, including project management, financial resources, government support, human resources, and organizational and technical requirements [93]. Furthermore, Duncan and colleagues proposed a seven‐dimensional framework for assessing HIS maturity: strategy, IT capability, interoperability, governance and management, patient‐centered care, people, skills and behavior, and data analytics [94]. These studies suggest that a comprehensive assessment of digital maturity must integrate technological, organizational, and human factors to ensure successful adoption and implementation of smart healthcare technologies.

### 4.2 Digital/Smart Hospital

A smart hospital can significantly improve patient safety, quality of care, affordability, and patient‐centered services, offering new value and insights to patients and clinicians that are not available in traditional medical services [22]. The findings of this study indicate that the effort to apply information technology in hospitals has led to the concept of a digital hospital, achieved through the use of PACS and electronic patient records to digitalize processes. Interoperability and digitalization of data and processes allow for data exchange and improve information accessibility.

IoT and AI‐enabled systems, as well as location tracking and identification technologies, could facilitate resource management, patient monitoring, and the delivery of integrated healthcare services. Robots, high‐speed communication, and telemedicine technologies for treatment or educational purposes make hospital services safer and more efficient. Furthermore, mobile and wireless networks make hospital operations more flexible, potentially transforming the traditional notion of a hospital in the future [95].

Phung et al. [96] suggested a pervasive healthcare solution that connects doctors, nurses, and other individuals through reliable mobile and wireless networks. They argued that the traditional concept of a hospital as a physical location for medical treatment might change, as pervasive healthcare enables more flexible and accessible options due to the widespread use and improved performance of wireless and mobile networks [96].

Smart hospitals should support digital network infrastructure and incorporate flexible and connected devices [97]. In a transition from a digital to a smart hospital, the IoT plays an essential role in connecting different devices using sensors, internet protocols, databases, cloud computing, and analytics [97]. Implementing the necessary policies both within and outside the hospital is essential for successful digital transformation.

Hospitals today must revisit their policies and strategies to update existing processes and physical spaces. Creating the technical infrastructure and providing an appropriate platform for developing smart hospitals are important. Within hospitals, both technical and organizational aspects require careful attention [82].

Regarding external hospital policies, governments need to establish effective incentive schemes to encourage hospitals to embrace digitalization, leading to optimized hospital processes and an expanded healthcare market. Governments can stimulate growth in the smart hospital industry ecosystem through various strategies, such as offering direct and indirect incentives, including certification through accreditation bodies [22]. In addition, the health sector needs to collaborate with industry sector to commercialize relevant technologies. Industry stakeholders, universities, and researchers, particularly in medicine, should form a standardization advisory body to lead standardization efforts domestically and internationally while boosting market competitiveness [22].

Introducing smart hospitals shifts the focus to continuous care, disease prevention, collaborative care, and patient involvement, emphasizing ongoing care over episodic treatment. Data collected in smart hospitals could help more effective policymaking and more efficient service delivery. In a technologically driven smart hospital, wireless communication is vital for information sharing inside and outside the hospital [95].

### 4.3 Challenges

The use of smart technologies has not been as successful as expected due to several challenges, issues, such as security, privacy, and spectrum congestion [96]. Enhancing information security and confidentiality is a top priority, and currently, there are no global standards for privacy and security policies. In Europe, cities like Barcelona and Amsterdam follow the General Data Protection Regulation, which is enforced by the European Union and focuses on data protection and privacy [97]. In contrast, cities like Edmonton and Drayton Valley in Canada adhere to the Freedom of Information and Protection of Privacy Act, which applies only in the province of Alberta [95].

Managing smart healthcare systems securely has become increasingly challenging because due to data security issues [98]. Ahmed and colleagues proposed using a hybrid communication system (radio and optical) to improve performance, adaptability, security, and comparability [99]. Blockchain technology, with its unique data storage method, offers enhanced security, accountability, adaptability, and authentication for data access can help protect healthcare data from certain threats [100, 101].

Developing a smart hospital is a complex process that requires a long‐term strategy, effective change management and the involvement of managers and stakeholders at different levels [4, 102]. The healthcare sector must build a robust digital health infrastructure that includes connectivity, secure data storage, and satisfactory data access and sharing. In addition, a strong governance framework is needed to support change management and foster a culture of digital transformation, including transparency in data ownership, cybersecurity, patient satisfaction, and patient education [103]. The lack of knowledge and expertise related to digital transformation presents a significant challenge for many healthcare workers in recommending digital services and products to patients. Therefore, digital literacy training for leaders, managers, and change agents is crucial to help them better understand and promote digital services and products [104].

### 4.4 Strengths and Limitations

This study provides a comprehensive overview of features and technologies that define digital and smart hospitals, providing valuable insights into the transition to smart healthcare facilities. It serves as a useful guide for hospital managers looking to upgrade their institutions and helps in making informed decisions for strategic improvements.

However, the study has two main limitations. First, a clearer distinction between features specific to each type of hospital would enhance precision. Despite this, the study effectively links these elements to the distinct objectives of digital and smart hospitals. Second, the focus on English‐language literature restricts access to a broader range of perspectives, potentially narrowing the study′s scope. For policy‐making applications, the findings need to be contextualized based on the macro‐ and micro‐determinants affecting the implementation of smart and digital hospitals.

## 5 Conclusion and Future Work

According to the findings of this study, the initial step in transitioning to a smart hospital is reshaping the organization's attitude, values, and strategy, alongside reengineering existing processes and addressing both organizational and technical factors. The introduction of new technologies inevitably alters workflows, making it essential to establish a clear strategy and revise care processes, management systems, and physical infrastructure to enable innovative models of care.

Implementing smart technologies such as mobile networks, wireless technology, remote monitoring systems, location tracking, IoT, blockchain, and AI requires digitization data and processes, as well as interoperability between systems. In this context, the digitization of services and the widespread use of EMRs are essential for building the foundation of a smart hospital.

Further research is recommended to explore the broader multifaceted implications of digital transformation in healthcare, including economic, ethical, social, legal, and clinical dimensions. Future studies should also focus on developing user‐friendly and cost‐effective implementation models given the high costs of these technologies, and assess how digital innovations influence patient outcomes, provider performance, and stakeholder engagement to evaluate their overall effectiveness.

## Author Contributions

**Reza Rabiei:** conceptualization, methodology, data curation, writing – review and editing, project administration. **Wen‐Chin Hsu:** conceptualization, data curation, writing – review and editing. **Peivand Bastani:** conceptualization, methodology, data curation, writing – review and editing, project administration. **Sohrab Almasi:** conceptualization, methodology, writing – original draft, data curation, writing – review and editing, visualization, project administration. **Saman Mortezaei:** writing – original draft, data curation, writing – review and editing. **Shirin Dehghan:** writing – original draft, data curation, writing – review and editing. All authors have read and approved the final version of the manuscript.

## Ethics Statement

The authors have nothing to report.

## Conflicts of Interest

The authors declare no conflicts of interest.

## 1 Transparency Statement

The lead author Reza Rabiei affirms that this manuscript is an honest, accurate, and transparent account of the study being reported; that no important aspects of the study have been omitted; and that any discrepancies from the study as planned (and, if relevant, registered) have been explained.

## Supporting information

S1_file.

S2_file.searchdocx.

S3_file.

## Data Availability

The authors confirm that the data supporting the findings of this study are available within the article. Also, data that support the findings of this study are available in the Supporting Material of this article. The corresponding author Sohrab Almasi had full access to all of the data in this study and takes complete responsibility for the integrity of the data and the accuracy of the data analysis.
