# Supplementary material for: Transition Toward Smart Hospitals: A Scoping Review of Features, Technologies, and Challenges
Source: Health Sci Rep. 2025 Nov 30;8(12):e71601. doi: 10.1002/hsr2.71601 (PMC12665508; doi:10.1002/hsr2.71601)
Supplement: Supplementary file 2 — S2_file.searchdocx. [file HSR2-8-e71601-s002.docx]

**Supplementary File 2. Search strategy for each database**

**Pubmed**

((“digital hospital” [Title/Abstract] OR "smart hospital" [Title/Abstract] OR "intelligent hospital" [Title/Abstract] OR "digital transformation" [Title/Abstract]) AND (characteristics[Title/Abstract] OR requirement [Title/Abstract] OR features [Title/Abstract] OR Challenges[Title/Abstract]))

**Scopus**

(TITLE-ABS("digital hospital") OR TITLE-ABS("smart hospital") OR TITLE-ABS("intelligent hospital") OR TITLE-ABS("digital transformation")) AND (TITLE-ABS(characteristic*) OR TITLE-ABS(requirement*) OR TITLE-ABS(feature*) OR TITLE-ABS(challenge*))

**IEEE**

(("All Metadata":"digital hospital" OR "All Metadata":"smart hospital" OR "All Metadata":"intelligent hospital" OR "All Metadata":"digital transformation") AND ("All Metadata":characteristics OR "All Metadata":requirement OR "All Metadata":features OR "All Metadata":challenges))

**Web of Science (WoS)**

TS=("digital hospital" OR "smart hospital" OR "intelligent hospital" OR "digital transformation")

AND TS=(characteristic* OR requirement* OR feature* OR challenge*)

**ScienceDirect**

("digital hospital" OR "smart hospital" OR "intelligent hospital" OR "digital transformation")

AND (characteristic* OR requirement* OR feature* OR challenge*)

**Wiley Online Library**

("digital hospital" OR "smart hospital" OR "intelligent hospital" OR "digital transformation")

AND (characteristic* OR requirement* OR feature* OR challenge*)
