# Supplementary material for: Transition Toward Smart Hospitals: A Scoping Review of Features, Technologies, and Challenges
Source: Health Sci Rep. 2025 Nov 30;8(12):e71601. doi: 10.1002/hsr2.71601 (PMC12665508; doi:10.1002/hsr2.71601)
Supplement: Supplementary file 3 — S3_file. [file HSR2-8-e71601-s001.docx]

Supplementary File 3**.** Characteristics of the included reviews.

| **Row** | **Authors, year, country** | **Type of Study** | **Aim/objective** | Main results |
| --- | --- | --- | --- | --- |
| 1 | Hruby et al., 1992, Germany | Case report | To overview of the SMZO-PACS-Project in the form of a rough specification of the system architecture and the functional parameters related to it. | -PACS modules are designed to minimize the workload  -PACS module includes image acquisition devices of a certain modality with related reporting workstations and a distributed electronic archive |
| 2 | Kuzmak and Dayhoff.,  1992, USA | Case report | To describe the components of the integrated DHCP imaging system and a commercial image A filing and communication system (PACS) works Concert to provide a wide range of department and Hospital-wide imaging capabilities. | -The ACR-NEMA message specification has been completed and prototype messages have been exchanged.  -The DHCP Imaging System can read and generate ACRNEMA messages.  -It can also convert and display images in ACR-NEMA format from the PACS. |
| 3 | Siegel et al., 1995, | Cross sectional study | Evaluation of the impact of picture archiving and communication system (PACS) on radiology productivity, timeliness of imaging reports, and rate of film loss and re-filming. | -The use of the PACS may also increase the efficiency of clinicians and other hospital staff which could result in substantial savings on a hospital-wide basis.  - improvements in radiologist productivity, timeliness of imaging reports, and film retake and film loss rates were recorded |
| 4 | Hruby et al., 1996, Austria | Case report | - To elaborate the key challenges and considerations in implementing a digital hospital information technology system in radiology? | - The implementation of PACS at the Danube Hospital was associated with a reduction in the average length of stay (ALS) for patients, which at 6 days was the lowest among all Austrian hospitals despite no difference in the medical spectrum.  - The PACS system reached a level of maturity and reliability that made it comfortable to use and a real support for the radiologists and other staff using it. |
| 5 | Takeda et al., 1998, Japan | Case report | - Develop an "intelligent hospital" with an Electronic Patient Record (EPR) system to replace conventional paper medical records  - Develop a Hospital Information System (HIS) called "HUMANE" to support the EPR system | - The Osaka University Medical School and its attached hospital planned to construct an "intelligent" hospital in 1993 to respond to growing needs for clinical, educational, and academic functions.  - The EPR system (EPROU) is currently under development and will be operational in a few years, and its development is closely connected to the quality and 6maintainability of the conventional medical charts management and the Hospital Information System (HIS). |
| 6 | Davidson et al., 1999, United States | Case study | - to develop a theoretical framework and to assess its usefulness for investigating linkages between technology design, organizational goals and, and users actions during system use on organizational outcomes. | - The introduction of the COE system led to changes in the content and structure of order-related communications between different occupational groups, creating ambiguity and the need for day-to-day problem solving.  - The COE system may be enabling increased organizational control over clinical care practices, through the development of protocols and guidelines, as well as physician profiling, though this was not seen as a concern by informants.  - Further research is needed on the consequences of structuring order-related communications in automated systems and the need to integrate the COE with other clinical systems to minimize disruptions. |
| 7 | Pavlopoulos et al. , 1999, Greece | Applied study | To develop and Install a DICOM-compliant PACS system at the Onassis Cardiosurgery Center (OCC) hospital | - The system enabled fast networking within the hospital to access patient files from anywhere, and integration with existing hospital information systems.  - The PACS reduced the time for examinations to become available to physicians and provided image processing tools to improve interpretation. |
| 8 | Liu et al., 2002, china | Observational study | - To design a digital health check-up system modeled on an industrial assembly line | - The digital assembly-line health check up is more efficient and productive than traditional random health check ups, taking less time, accommodating more customers, and improving staff competence and quality while reducing costs.  - However, the digital assembly-line approach is inflexible and cannot easily adapt to changing customer demands, requires significant investment to adjust, and may lead to low-skill, monotonous work for staff. |
| 9 | Chang et al., 2003, China | Observational study | -To enable telemedicine and real-time information sharing between radiologists and clinicians and provide individualized patient services through a large patient database | - The paper describes a digital hospital information system that integrates HIS, RIS, and PACS through a modularized structure following international standards, providing advantages such as common sharing, openness, security, extensibility, and simple operation.  - The integrated system has been successfully implemented in many hospitals in China, leading to benefits such as reduced diagnosis duration, improved work efficiency and medical level, and increased revenue.  10- The system realizes the seamless connection among HIS, RIS and PACS through modularized interfaces, achieving system function, management function, information processing and communication function on a complete platform. |
| 10 | Hanping et al., 2004, China | Observational study | -Integrating regional health resources (human resources, information technologies, funds, experiences, project management) to establish a digital hospital | - Digitized hospitals are important for reducing medical errors, improving quality of care, and lowering costs.  - Information technology has great potential to reconstruct the public health service system.  - Establishing a digital hospital is a long, challenging, and complicated process that requires collaborative efforts and utilization of various resources. |
| 11 | Lee et al. , 2007, Korea | Observational study | To address the security and privacy issues that arise from using RFID technology in medical and healthcare environments | - The authors proposed a privacy architecture for RFID-based medical applications, including a Privacy Controlled Data (PCD) scheme for transferring medical data between hospitals and a discovery gateway to connect to the privacy-controlled medical system.  - The authors state that this proposed mechanism is an effective solution for privacy control in RFID-based hospital systems. |
| 12 | Coronato et al., 2008, Italy | Applied study | - To develop semantic models, mechanisms and services to locate mobile objects using diverse positioning systems | - The paper presents a semantic location service that uses ontologies and rules to define a uniform model for location information, independent of the positioning system used.  - The service performs reasoning to provide physical and semantic locations of mobile objects, and to give the location information with the finest granularity when an object is located by multiple positioning systems.  - This allows the environment to customize services based on user location, and enables mobile users to access services. |
| 13 | Wu et al, 2005, China | Applied study | - Develop an event-based architectural solution (e-Wellness) to manage and process RFID sensor data in hospitals | - The paper proposes an "edge ware" architecture called e-Wellness to manage and process RFID sensor data in a hospital environment, with the goal of optimizing hospital processes.  - The key aspect of this architecture is the use of an "event" as the fundamental unit for modeling and reasoning about the sensor data, which allows handling large volumes of heterogeneous data in real-time.  - The paper describes a three-layered event representation and reasoning model to process the sensor data, going from low-level data events to higher-level domain events. |
| 14 | Huang et al., 2008, China | Applied study | - Integrate RFID technology with existing Hospital Information Systems (HIS) to Improve the efficiency of hospital management and patient safety in medication use and medical care | - RFID technology can be used to improve the operational efficiency and patient safety in healthcare facilities.  - RFID tags can be embedded in patient ID bracelets, charts, and medical equipment to automatically track their location and status. - RFID technology can be integrated with existing hospital information systems to improve the efficiency of hospital management and patient safety, particularly in medication use and medical care. |
| 15 | Coronato et al., 2008, Italy | Applied study | - Develop a semantic location service that can integrate and reason on location information from multiple positioning systems | - The location service integrates different positioning systems and uses semantic web technologies to provide a uniform representation of location information and enable reasoning on this information.  - The location service can provide both physical and semantic locations of mobile objects, and can infer the most granular location when an object is detected by multiple positioning systems.  - The location service uses a homogeneous approach to integrate ontologies and rules, translating them into a common logical language and using a single inference engine for reasoning. |
| 16 | Hu et al., 2010,  China | Document analysis | - | - Integrating information across the value chain (patients, hospitals, suppliers) and integrating human, knowledge, management, and strategic objectives can improve hospital operational effectiveness and competitiveness.  - The business application system of a digital hospital should be centered around a medical service platform, integrated with other platforms through data centers and integration platforms, and connected to external systems, forming a comprehensive digital information platform. |
| 17 | Yao, 2011, singapore | Feasibility study | - Seek a viable approach to improve patient safety through proposing RFID-enabled CEP framework  - Answer questions about challenges faced in hospitals, how RFID can help, what CEP is and how it can be used, and the expected benefits of an RFID-enabled CEP framework in hospitals | - The proposed RFID-enabled CEP framework provides a feasible solution to improve patient safety and operational efficiency in hospitals by detecting medically significant events in real-time.  - The performance evaluation shows that the approach has reasonable processing delay and detection accuracy.  - The proposed solution aims to improve patient safety and reduce operational costs in hospitals, particularly for problems encountered during surgical procedures. |
| 18 | Vecchia, 2012, Italy | Development and evaluation Study | - Introduce an intelligent infrastructure for smart hospitals | - An intelligent software/hardware infrastructure is presented which is intended to shorten the patent gap existing between healthcare domain constraints and pervasive enabling technologies.  -The rationale of this infrastructure’s design derives from the need to simplify a context-aware access to the underlying environmental resources of a typical healthcare facility while keeping flexibility and extendibility at a reasonable level |
| 19 | Yoo, et al., 2012, Korea | Observational study | -Introduce the major components of the SNUBH information system  -Describe the progress toward a next-generation hospital information system (HIS) | - SNUBH has adopted innovative health information technology to improve the efficiency and quality of patient care.  - SNUBH was the first hospital outside of North America to achieve Stage 7 accreditation for its comprehensive EHR system.  - SNUBH is currently developing a next-generation hospital information system (HIS) with goals of implementing flexible and innovative infrastructure, a patient-centered system, and strengthening IT capabilities to maximize hospital value. |
| 20 | Peleato, t al., 2013 | Observational study | - Propose an innovative solution for the localization of autonomous delivery robots in hospitals - Develop a Smart Global Positioning System (S-GPS) framework and a novel algorithm for tracking delivery robots using multi-lateration with an optimal number of references | - The authors propose an innovative S-GPS framework and a novel multi-lateration algorithm to accurately localize delivery robots in hospitals.  - The S-GPS framework can compute coordinates of all static and mobile sensors and is fault-tolerant.  - The multi-lateration algorithm can localize the robots to within 3 meters of error using an optimal number of 31 reference nodes. |
| 21 | Alharbe et al., 2014, United kingdom | Mixed-method study | -To develop a comprehensive real-time tracking and monitoring system to improve healthcare services | - A combination of RFID and ZigBee technologies can provide a solution for monitoring objects, including patients and staff, in the hospital.  - The survey participants, especially nurses and doctors, strongly agreed on the benefits of implementing an automated tracking system, including improved real-time information access, increased productivity, and improved healthcare quality.  - A majority of the participants, including 58% of urses, 62% of doctors, 40% of administrators, and 100% of ancillary staff, strongly supported the implementation of an automatic monitoring system. |
|  | Mahmood et al., 2014, Malasia | Development Study | -Identifying the key entities in smart hospital system and developing the database model (ERM) by integrating RFID technology.  -Proposing architecture for RFID based smart hospital systems, developing a working prototype application. | - RFID systems are integrated into hospital information systems and provide full automation and streamline the important modules of patient identification, staff allocation, doctors, medicines and treatments. |
|  | Park et al., 2014, Korea | Retrospective observational study | - To describe the lessons learned from developing 12 health apps in a large tertiary hospital in Korea - To promote the use and adoption of health apps for patient care by describing the hospital's experience | - The choice of operating system (OS) for health apps should be based on market share or the hospital's strategic plan.  - Smartphones are generally preferred over tablets as the target device for health apps.  - Developing health apps through an alliance with an IT company is the best approach. |
|  | Thakare et al., 2014, India | Descriptive, observational | - Analyze the current hospital information system  - Identify key areas for improvement in the current hospital information system  - Identify newer/cutting-edge technologies that can be used to improve the hospital information system | - Transforming hospital information systems into "smart" systems by leveraging emerging technologies can improve the delivery of healthcare services and make processes more efficient.  - Integrating various hospital systems and enabling seamless data flow can help deliver better care and enhance satisfaction for patients, care providers, and attendants.  - Adopting new technologies like tablets, digital medical equipment, and smart applications can improve workflow, enable remote monitoring, and enhance operational efficiencies in the hospital. |
|  | Aktepe, et al., 2015, Turkey | Development Study | - Apply an expert system on a web application to direct patients to the right department | - The internet-based expert hospital appointment system helps users choose the right outpatient department/polyclinic.  - The system provides benefits such as shorter waiting times in hospitals, decreased number of consultations, and time savings for both patients and doctors.  - The system helps standardize service rates in outpatient departments/polyclinics. |
| 22 | Alharbe et al., 2015, saudi Arabia | Development and Evaluation Study | Propose a smart monitoring system using RFID and ZigBee technology to detect, locate, and monitor the movement of objects in a hospital | - The paper proposes a smart monitoring system that integrates cloud computing, wireless sensor technology (RFID and ZigBee), and the Internet of Things to continuously detect, locate, and monitor the movement of objects (patients, staff, equipment) in a hospital environment.  - The system consists of a coordinator node to acquire object information at the data collection layer. |
| 23 | Gomez, et al., 2015, Spain | Simulation study | - Evaluate the quality of service in communications for a smart hospital setting with heterogeneous services | - Designing external communications for a smart hospital is a complex task due to heterogeneous services and evolving technology, but simulation tools can provide flexibility in analyzing scenarios with HTC and MTC sources.  - The authors designed a connectivity scenario, simulated it in a real operator environment, and adjusted parameters to ensure quality for HTC services, while MTC services in a lower quality class experienced significant delays and losses. |
| 24 | Vazquez-Santacruz et al., 2015,  Mexico | Applied study | - Develop an intelligent system to detect patient posture on a hospital bed using a mattress pressure sensor | - The authors developed an intelligent system that can detect the posture of patients on a robotic hospital bed in real-time with high accuracy (99.01%).  - This intelligent system can be used to monitor the patient's position and prevent accidents when the bed is moving, by stopping or adjusting the bed's movement if an inappropriate posture is detected.  - The system is able to process 100 sensor arrays per second, which matches the hardware's capabilities, allowing for real-time monitoring. |
| 25 | Venkateswari et al., 2015 | Development study | -Develop a wireless body area network (WBAN) to continuously monitor mobile patients  - Efficiently route the monitored information to the sink  - Optimize RPL and MAC parameters to address the challenges of increased power consumption and transmission range due to mobility | - The proposed optimized RPL protocol parameter values provide a high packet delivery ratio of 98% for a mobile WBAN environment, with only a slight increase in power consumption compared to default values.  - The optimized parameter values resulted in twice the packet delivery ratio compared to the default values, while maintaining a tolerable power consumption of 5 mW.  - The authors state that this design is appropriate for a mobile WBAN environment for patient monitoring in a smart hospital scenario. |
| 26 | Alharbe et al., 2016, United kingdom | Development and evaluation study | - Develop an automatic real-time data collection system to continuously monitor patients, staff, and assets using sensor technologies | - The proposed Smart Hospital Management Information System (SHMIS) uses RFID and ZigBee technologies to continuously monitor and track patients, staff, and assets in real-time.  - The SHMIS provides decision support for healthcare professionals by integrating sensor data with knowledge harvested from the Communities of Practice, improving operational and staff performance.  - The SHMIS aims to transform the manual hospital systems currently in use into a smart, automated system that can provide real-time information and decision support. |
| 27 | Yoo et al., 2016, South Korea | Retrospective study | To share the experiences and achievements of implementing hospital information systems and electronic health record (EHR) systems at the Seoul National University Bundang Hospital (SNUBH) over 13 years. | The main findings include the comprehensive nature of SNUBH's HIS in providing care across various hospital units, the emphasis on user experience design methodology in system development, and the importance of the CDW system for big data analytics in healthcare research. |
| 28 | Rizwan et al, 2017, India | Development and evaluation study | presents an innovative technical support for development of smart hospitals with low investment | -Patient remote monitoring system monitors the chronic disease patient’s health condition continuously and generates alerts during abnormal situations of patient’s health  -A Patient remote monitoring system includes wearable devices which are developed by using Internet of Things. The wearable devices track the patients’ health condition continuously |
| 29 | Chen et al., 2018, Tiwan | Development and evaluation study | - Develop a low-cost Wi-Fi RSSI-based indoor positioning system (IPS) for a smart hospital environment - Estimate unknown locations by evaluating RSSI and MAC addresses | - The proposed Wi-Fi RSSI-based indoor positioning system achieves an error distance of about 2 meters and nearly zero mislocations for room identification.  - The system uses low-cost hardware components like an Arduino platform, RedBearLab CC3200 WiFi Mini module, Li-ion battery, speaker, OLED display, and cloud server.  - Experimental results demonstrated a positioning error of around 2 meters in a large hospital building. |
| 30 | Pham et al., 2018, | Development study | - Develop a remote healthcare system using blockchain and IoT technology to address privacy and security concerns | - The authors created a remote healthcare system using blockchain to manage patient information and medical devices.  - The system automatically writes patient health data from sensors into the blockchain. - The authors proposed a mechanism to efficiently store medical device information by only writing abnormal sensor data to the blockchain, reducing the blockchain size and transaction costs. |
| 31 | Zhang et al., 2018 | Observational study | - Formalize an architecture using NB-IoT to connect all intelligent things in smart hospitals - Design an infusion monitoring device based on infrared sensors and connect it using NB-IoT | - The authors proposed an architecture based on NB-IoT to connect intelligent devices in smart hospitals, taking advantage of NB-IoT's higher capacity, wider coverage, lower power consumption, and lower cost.  - As a case study, the authors designed an infusion monitoring device using infrared sensors and NB-IoT, which can accurately count drops even in sunlight interference, and used a fault detection process and learning algorithm to calculate the remaining drug volume. |
| 32 | Barnett et al., 2019, Australia | Case study | The aim of the study was to deliver safety and quality data in near real time to the hospital executive and clinicians to facilitate better patient care. | - The use of real-time digital dashboards for hospital quality and safety standards allowed clinicians and managers to identify issues and intervene in a timely manner.  - The analytics were associated with increased compliance with the recommended standards, though it was too early to claim large-scale improvements, as the insights from the data still needed to be acted upon.  - This was the first known implementation of a live streaming clinical analytics platform to improve quality and efficiency across the 10 national hospital standards. |
| 33 | Fischer et al., 2019, Brazil | Development and evaluation study | - Devise Human Resources IoT-based Elasticity for automatic management of human resources in healthcare environments | - The ElHealth model was able to significantly decrease patient waiting times in a simulated hospital environment, by up to 96.4% using a reactive approach and 96.73% using a proactive approach.  - The authors expect that the ElHealth model can help decrease patient waiting times in real-world healthcare settings.  - The case study results show that ElHealth achieved substantial reductions in patient waiting times compared to the baseline scenario without the model. |
| 34 | Huang, et al., 2019, China | Development study | - To develop an intelligent hospital system that is centered on patients and uses the internet and big data to provide humanized services for both medical staff and patients | - Blockchain technology is used to establish a decentralized and transparent data mart for intelligent hospitals, with each data block being immutable and authorized.  - Blockchain technology combined with homomorphic encryption and zero-knowledge proof is used to protect patient privacy, by storing patient data in an encrypted "DNA wallet" and only revealing the final diagnosis result without exposing the underlying data.  - The paper also discusses other security measures for intelligent hospital systems, such as prohibiting external network access and regularly updating systems. |
| 35 | Thangaraj et al., 2015, India | Development and evaluation study | - Describe the implementation of a smart autonomous hospital management system using IoT technology - Explain the technology drivers behind IoT and healthcare | - The smart hospital management system allows hospital authorities to connect medical devices, define data models, data mapping formats, and workflows/processes with configurable roles and authentication.  - The system can integrate with existing and external systems, and supports defining data formats at different levels (raw, processed) to adhere to medical record standards.  - The system faces challenges with limited battery life in devices, which requires a power management framework and routine device validation. |
| 38 | Jamil et al., 2020, South Korea | Development and evaluation study | - Develop a decentralized healthcare IoT platform based on permissioned blockchain to address challenges like data security, identity, and scalability | - The proposed system is scalable and can handle numerous healthcare IoT devices connected through different networks to a single blockchain.  - The use of a permissioned blockchain with authenticated/registered entities improves the throughput of the network by enabling the use of fault-tolerant protocols.  - The proposed system is transparent as it hides information of IoT devices and transaction logs from unauthorized users. |
| 39 | Konig et al., 2019, Germany | Retrospective observational study | Implement a knowledge-based "best of breed" approach combining a terminology server, NLP pipeline, and rules engine | - The system was able to detect osteoporosis with 94.19% precision and 97.45% recall.  - The system was able to detect proton-pump inhibitors (PPIs) with 100% precision and 97.97% recall.  - The overall F-score for detecting the drug-disease interaction of PPI use and osteoporosis was 96.13%. |
| 40 | Kumar et al., 2019, India | Case report | proposing a novel model which focuses on a smart hospital information management system that runs by using hybrid cloud, IoT, ML, and AI. | -Patients and doctors unique ID would make the entire process a lot more efficient and easier. The advances happening in the field of AI and ML due to cloud-based computing is extremely beneficial for the medical industry.  -By integrating these components along with IoT it is possible for multi-specialty hospitals and super specialty hospital to be able to set up a smart hospital information management system. |
| 41 | Scott et al., 2018, Australia | Observational case study | The objective of the study was to develop a checklist that clearly and comprehensively defines the steps that best prepare hospitals for EMR implementation and digital transformation. | - The paper developed a 19-question checklist to guide hospitals in preparing for electronic medical record (EMR) implementation and digital transformation. - The checklist covers key considerations for both EMR implementation and digital transformation.  - The checklist was developed through a formal methodological framework including literature review, a workshop with digital leads, and review by clinical leads. |
| 42 | Kim et al., 2020, South Korea | Case Study | To implement digital solutions effectively to improve patient safety, medical service, and management efficiency | - Yongin Severance Hospital has set three main goals for establishing an efficient and effective digital hospital: patient safety and convenience, high-quality medical service, and efficient management.  - Yongin Severance Hospital has developed various digital solutions to achieve these goals, such as an integration and response space (IRS), real-time location systems (RTLSs), facial recognition systems, and a mobile app for patients.  - Yongin Severance Hospital has established a "Digital Medical Industry Center" to collaborate with industry, academia, and research institutes to develop and manage digital solutions, and to create a new medical ecosystem. |
| 43 | Williams et al., 2019, United states& Australia | Development and evaluation study | - To develop a framework to assess the maturity of a hospital's information technology infrastructure  - To enable hospitals to benchmark their current infrastructure capabilities and create a vision for future infrastructure development  - To provide hospital administrators and clinicians with guidance on how and why to invest in information infrastructure to support health IT that benefits patient safety and care | - The Infrastructure Maturity Assessment (IMA) framework defines 164 individual capabilities across 5 technology domains and 8 maturity levels to characterize a hospital's information infrastructure and support digital transformation.  - The IMA framework provides a tool to assess a hospital's preparedness to support existing or planned digital processes by classifying the sophistication of their information processes.  - The IMA framework articulates how hospitals can generate more value from their infrastructure by defining the levels at which critical "enabling information" characteristics are delivered, and the ICT infrastructure requirements to achieve those levels of experience. |
| 44 | Rajakumari et al. , 2020, India | Development and evaluation study | -Develop a web-based appointment system to improve efficiency, quality, and reduce patient waiting time  - Use a Convolutional Neural Network (CNN) for clinician schedule analysis and optimization | - The paper proposes a Convolutional Neural Network (CNN) based system to optimize clinician scheduling and reduce patient waiting times.  - The system is built on process mining analysis, including process discovery, patient arrival rate analysis, and service time analysis.  - The system includes steps to determine the optimal improvement strategies to reduce waiting times. |
| 45 | Krasuska et al., 2019, Mulinational | Qualitative analysis | To develop an international agreement on a defined set of technological capabilities to assess digital excellence in hospitals | - The authors have identified a set of technological capabilities that can be used to assess digital excellence in hospitals.  - The authors question the appropriateness of stage-based models for assessing digital maturity and highlight the need to consider the integration of digital capabilities across the broader healthcare ecosystem. |
| 46 | Soman et al., 2020, India | Development study | - Develop a mobile-augmented smart queue management system (QMS) that can be integrated with a hospital's existing Hospital Management Information System (HMIS) to provide end-to-end patient queue management across various service areas in the hospital. | - The QMS allows the hospital administrator to configure and manage the service-based counters, and incorporates smart algorithms for dynamic token generation and allocation to streamline patient experience and manage high patient loads.  - The deployment of the QMS enables the hospital to monitor the performance of the service counters and service areas, and track key performance indicators (KPIs) related to the patient workflow, which can be used for resource planning and optimization. |
| 47 | Uslu et al., 2020, Germany | Development study | - Propose a five-layered IoT architecture that can efficiently utilize the above factors - Explain and compare key technologies related to each layer and their impact on IoT architecture design  - Discuss possible future directions and research challenges | - It identifies the optimization factors, challenges, available technologies, and opportunities for employing IoT technology in smart hospital environments.  - It explains the deficiencies that may arise in each layer of the smart hospital design model and the factors that should be considered to address them.  - It provides a roadmap for managers, system developers, and researchers interested in optimizing the design of smart hospital systems. |
| 48 | Abdulkareem et al., 2021, Brazil | Case report | - To propose a model based on machine learning (ML) and Internet of Things (IoT) to diagnose patients with COVID-19 in smart hospitals. | - The SVM classifier achieved the best COVID-19 prediction performance, with an accuracy of up to 95%, precision of 95%, recall of 95%, and F1-score of 95% on the normalized dataset with brute-force feature selection.  - The SVM classifier also performed well on the original dataset, with an accuracy of 93.33%, precision of 94%, recall of 93%, and F1-score of 92%.  - The use of normalization and feature selection techniques further improved the performance of the SVM classifier. |
| 49 | Akbarzadeh et al., 2021, Iran | Cross sectional study | - Designing and developing an innovative solution for smart buildings to increase hospitality during COVID-19 and future pandemics | - The main findings of this paper are the development of an IoT-based smart building solution that can address issues like occupancy control, smart navigation, and queue management during the COVID-19 pandemic, and the development of a novel IoT-based platform that can be implemented in real-world projects and a mobile application to provide the services to end-users.  - The proposed solution provides practical information to end-users, such as queue management, navigation, and social distancing notifications, which are particularly useful during the COVID-19 pandemic. |
| 50 | Jang et al., 2021, Korea | Development study | - Propose a group number-based WSN that can relay data to the AP without requiring direct connection, to ensure network connectivity for both fixed and mobile sensors | - It proposes a method to deploy access points (APs) in a hospital sensor network that minimizes the overall network operation cost. - It assigns group numbers to sensor nodes to guarantee network connectivity, even when mobile sensor devices move around.  - The proposed method is more efficient in terms of computation time compared to a brute-force approach or a genetic algorithm. |
| 51 | Mezenner et al., 2021, Algeria | Development study | - Develop a WoT-based healthcare system for hospitals to monitor patients' vital signs remotely  - Provide automatic intervention scenarios to treat patients remotely | - The proposed WoT-based healthcare system can be beneficial during health crises and pandemics by reducing the workload on medical staff, decreasing costs, and improving the quality of services and user experience in hospitals.  - Several challenges need to be addressed to further improve the performance of the system, including maintaining data security and privacy, deploying efficient data management techniques, and constantly auditing system performance to prevent crashes. |
| 52 | Ravali et al., 2021, India | Development and evaluation study | -Design a patient health monitoring system  - Develop modules to promote physician diagnosis via patient telemonitoring | - The proposed system is well-integrated and takes advantage of advancements in integrated circuits and MEMS technology.  - The system is power-efficient and can quickly process data.  - The system can identify patients and continuously monitor them, and it provides power-efficient remote monitoring of patients and can help handle emergency cases immediately. |
| 53 | Rodrigues et al., 2021, Brazil | Development and evaluation study | -Develop an SPN model to evaluate the performance of smart hospital computing architectures  -Develop an SPN model to assess the availability of smart hospital computing architectures | - The proposed SPN model allows for the configuration of 13 parameters, enabling the evaluation of many different scenarios for smart hospital systems.  - The analysis showed that the arrival rate is an essential parameter, and there is a close relationship between mean response time, resource utilization, and discard rate, especially for high arrival rates.  - The scenario with server redundancy (local and remote) presented the highest availability at 99.9199%, with only 7.01 hours of inactivity per year. |
| 54 | Rosen et al., 2021, multinational | Development and evaluation study | To expand home telehealth to provide comprehensive medical care across a distributed network of hospitals and homes | - The paper recommends expanding home telehealth to provide comprehensive medical care across a distributed network of hospitals and homes, linking patients to healthcare workers through the Internet of Medical Things using in-home equipment and smart medical monitoring devices.  - The paper provides examples of current models of telehealth care in the home, including telehospital medicine, home-based care for chronic conditions, and hospital-at-home programs.  - The paper discusses the necessary baseline requirements for implementing a home telehealth model, including robust bandwidth, stable broadband internet, electrical backup, and various medical monitoring devices. |
| 55 | Yamashita, et al., 2021, Japan | Multi-part experimental study | - Evaluate the positioning accuracy of geomagnetic indoor positioning in hospitals  - Visualize the movements of people and goods in the hospital to improve operational efficiency | - The most accurate indoor positioning method was the combination of geomagnetic and Bluetooth Low Energy (BLE) beacons, with an average position error of approximately 1.2 m.  - Geomagnetic positioning alone had variable accuracy, but combining it with Wi-Fi or BLE beacons improved the positioning accuracy.  - The placement of BLE beacons was important for achieving stable positioning accuracy, with beacons installed at a height of 2 m in corridors and nurse stations performing better than those installed at 3 m in hospital rooms. |
| 56 | Atta, 2022, Saudi Arabia | Development and evaluation study | - To develop a cost-effective, reliable, and low-power wireless vital signs monitoring system for large hospitals | - The study developed a cost-effective, reliable, and low-power wireless vital signs monitoring system for large hospitals.  - The system was able to cover a 2500 m2 area with a low packet loss rate of less than 3.3%.  - The use of standard components and similar voltage sources improved the system's cost, compatibility, and simplicity. |
| 57 | Jebamani et al, 2022, India | Development and evaluation study | -To develp an android app to hospital room booking | -The Online Hospital Room Booking System provides people all over the world with an easy and fast way to book hospital rooms online when there is an emergency.  -Users can Perform room reservation activities anywhere by accessing them over the Internet. |
| 58 | Sakthikumar et al, 2022, India | Development and evaluation study | -To create an Android app with emergency medical, healping people search hospitals with the availability of doctors near them | -Our project helps the public to know doctors' availability near their locality. A mobile application that tracks the availability of doctors nearby the user's locality in times of emergency. After tracking the nearby hospital, if there is a need for an ambulance service, we can approach and track the ambulance through the proposed mobile application using IoT.  -The app also suggests a specific hospital for a specific ailment. The mobile application receives data from biometrics available in hospitals for attendance. With the help of those data, users will receive information regarding the doctor's availability. |
| 59 | Pan et al, 2023, USA | Development and evaluation study | -To design and implementation of a novel hybrid wireless tracking system utilizing visible light communication (VLC) and positioning (VLP) technologies, aided by powerline communication (PLC), for smart hospital operations | -Utilizing the existing LED lighting infrastructure, this VLC/VLP/PLC positioning system consists of host optical transceivers embedded in light-emitting diode (LED) bulbs and user-end photodetector (PD) optical tags to form a wireless tracking network connecting all people and equipment throughout all hospital buildings. |
| 60 | Talib, et al.,2023, Iraq | Development and evaluation study | - Developing a smart hospital system based on cloud computing and home automation to improve patient comfort and care | - The paper presents a smart hospital system based on cloud computing that can monitor patient vital signs such as body temperature, heart rate, and blood oxygen levels in real-time.  - The system uses IoT sensors and devices to collect patient data and transmit it to a cloud server for storage and processing.  - The system can alert healthcare providers if a patient's vital signs fall outside of normal ranges, allowing for early intervention. |
| 61 | Mi, et al.,2023, China | Retrospective cohort study | -To address the challenges posed by the COVID-19 pandemic, our hospital developed an intelligent hospital management mode specifically tailored to COVID-19 patients | -The development of intelligent management mode can reduce the burden of medical personnel and the probability of developing infection and bring about timely and better patient care.  -Intelligent management can play a pivotal role in control of the epidemic, treating patients, allocation of resources, tracing the root cause of the virus, and monitoring |
| 62 | Wosny, et al., 2024  Switzerland | Qualitative Study | -The goal of this study was to comprehensively explore the lived experiences of HCPs navigating digital tools throughout their daily routines. | -  The promise of efficiency and the reality of inefficiency, the shift from face to face to interface, juggling frustration and dedication, the illusion of information access and trust, the complexity and intersection of workflows and care paths, and the opportunities and challenges of shadow IT |
| 63 | Hughes, et al., 2024  Ireland | Qualitative Study | -The aim of this phase of a larger study was to explore the perspectives and opinions of key stakeholders on the requirements, benefits, and challenges for a bespoke patient portal, with a specific focus on the ANP-led Neurosurgical Service and children and young people with hydrocephalus. | -  More timely access to health data as well as a consistent log of information and communications between patients and healthcare professionals, would be more efficient and effective than current practices |
| 64 | Isakov, et al., 2024  Finland | Qualitative Study | - To describe healthcare providers' perspectives of digital transformation in hospital-at-home care. | 1) Health information exchange in and across hospital-at-home care; 2) Management of hospital-at-home care; 3) Logistics in hospital-at-home care planning and delivery; and 4) Digital health interventions in hospital-at-home care delivery |
